# A new measure for functional similarity of gene products based on Gene Ontology

**DOI:** 10.1186/1471-2105-7-302

**Published:** 2006-06-15

**Authors:** Andreas Schlicker, Francisco S Domingues, Jörg Rahnenführer, Thomas Lengauer

**Affiliations:** 1 Department of Computational Biology and Applied Algorithmics, Max-Planck-Institute for Informatics, Stuhlsatzenhausweg 85, 66123 Saarbrücken, Germany

## Abstract

**Background:**

Gene Ontology (GO) is a standard vocabulary of functional terms and allows for coherent annotation of gene products. These annotations provide a basis for new methods that compare gene products regarding their molecular function and biological role.

**Results:**

We present a new method for comparing sets of GO terms and for assessing the functional similarity of gene products. The method relies on two semantic similarity measures; *sim*_*Rel *_and *funSim*. One measure (*sim*_*Rel*_) is applied in the comparison of the biological processes found in different groups of organisms. The other measure (*funSim*) is used to find functionally related gene products within the same or between different genomes. Results indicate that the method, in addition to being in good agreement with established sequence similarity approaches, also provides a means for the identification of functionally related proteins independent of evolutionary relationships. The method is also applied to estimating functional similarity between all proteins in Saccharomyces cerevisiae and to visualizing the molecular function space of yeast in a map of the functional space. A similar approach is used to visualize the functional relationships between protein families.

**Conclusion:**

The approach enables the comparison of the underlying molecular biology of different taxonomic groups and provides a new comparative genomics tool identifying functionally related gene products independent of homology. The proposed map of the functional space provides a new global view on the functional relationships between gene products or protein families.

## Background

Genome annotation relies heavily on bioinformatics methods. The identification of homologous relationships is a powerful and frequently used approach for protein-level annotation [1], where query protein sequences are compared to sequences of characterized proteins in order to find homologies. Based on this comparison, proteins of unknown function are assigned to characterized protein families, generating testable hypotheses of their molecular function. However, this established annotation approach has several limitations. Devos and Valencia [2,3] suggest that up to 30% of the function annotations made through sequence similarity searches might be erroneous. Obviously, there is no simple relationship between sequence similarity and function, but some general trends have been observed. The same authors showed that the Enzyme Classification (EC) [4] number tends to be completely conserved only for proteins with more than 80% sequence identity. They found that it is problematic to assign EC numbers based on a sequence alignment with less than 30% identity.

Complementary to sequence similarity searches, more direct approaches for the functional characterization of gene products have been proposed. In particular, genomic context methods predict which gene products are involved in common biological processes [5,6]. Other methods use different protein features or structural information to predict the function of a gene product [7-9].

The Gene Ontology Consortium provides a structured standard vocabulary for describing the function of gene products [10]. The Gene Ontology (GO) is divided into three orthogonal ontologies, *biological process*, *molecular function*, and *cellular component*. The three ontologies are represented as directed acyclic graphs (DAG) in which nodes correspond to terms and their relationships are represented by edges. Each node can have several parents and several children. There are two types of relationships. "is-a" indicates that the child is a subclass of the parent, and "part-of" is used when the child is a component of the parent. GO terms are widely used to annotate genes and their products with functional terms [11].

New methods can exploit these GO annotations in order to compare gene products on the basis of their function. There are some issues which one has to take into account when GO annotations are compared. One problem is that the depth of a term in the GO graph is not representative of the specificity of the underlying concept. Different terms on the same rank (same depth in the GO graph) usually are not equally specific. In addition, GO is an ongoing project in which new terms are added continuously but many specific functional terms may still be missing. The manual mapping of GO terms to genes is based on results available in the scientific literature or in public databases, but relies on human decision and therefore is considerably subjective [12]. In addition, a large part of gene products is not yet annotated with GO terms. These problems have to be considered when designing robust measures to assess the similarity of two GO terms.

Semantic similarity measures have been proposed for comparing concepts within an ontology. Resnik [13,14] developed a measure of semantic similarity for "is-a" ontologies based on the information content of the lowest common ancestor (LCA) of two terms. The more frequently a term occurs, i.e., the higher its probability of occurring, the lower its information content. If the LCA of two terms describes a generic concept, these terms are not very similar and this is reflected in a low information content of their LCA. This measure considers how specific the LCA of the two terms is but disregards how far away the two terms are from their LCA. Lin [15] developed a related measure that depends on the information content of the LCA and of the two terms that are compared. This measure assesses how close the terms are to their LCA. It does not refect the level of detail of the lowest common ancestor, though.

Protein sequences annotated with GO terms can be compared on the basis of such semantic similarity measures. Lord *et al*. [16] were the first to apply a measure of semantic similarity to GO annotations. They implemented GOGraph, a tool for calculating the semantic similarity of protein pairs based on Resnik's measure. The semantic similarity between two proteins is defined as the average similarity of all GO terms with which these proteins are annotated. Each protein pair receives three similarity values, one for each ontology. Cao *et al*. [17] integrated a semantic similarity search into the Bio-Data Warehouse. They use also Resnik's measure to define the similarity between two single GO terms. Speer *et al*. [18] employed a distance measure based on Lin's similarity for clustering genes on a microarray according to their function. Khatri and Draghici reviewed tools for ontological analysis of gene expression data [19]. Friedberg and Godzik [20] used the molecular function annotation of protein structures in the Protein Data Bank (PDB) [21] to perform a functional comparison of different folds. They define a GO-based fold similarity as the normalized average Resnik term similarity of two folds. Lee and Lee [22] applied Resnik's semantic similarity measure to MIPS [23] and GO annotations in order to infer modularized gene networks. They divide the GO annotations into three sets, set 1 contains all GO terms annotated to both genes, set 2 and set 3 contain the GO terms annotated to only one of them. Then the maximum similarity between any terms from set 2 and terms from set 3 is calculated (*max*_2, 3_). Finally, the annotation information score is the sum of all self-similarities of terms in set 1 plus *max*_2, 3_. Shalgi *et al*. utilized Lord's definition for a subcellular clustering score based on the cellular component ontology. They calculate the similarity of two genes as the maximum similarity of GO terms annotated to one of the genes. Björklund *et al*. [24] developed a domain distance score for assessing the similarity of two domain architectures. They showed that the domain distance correlates well with Lord's approach to semantic similarity of proteins. Sevilla *et al*. [25] analyzed the correlation between gene expression and Resnik's and Lin's measures of semantic similarity. They concluded that Resnik's measure correlates well with gene expression.

Gene products are functionally similar if they have comparable molecular functions and are involved in similar biological processes. These gene products did not necessarily evolve from a common ancestor and therefore do not necessarily show sequence similarity. GO annotations capture the available functional information of a gene product and can be used as a basis for defining a measure of functional similarity between gene products. In this paper, we introduce a new measure of similarity between GO terms that is based on Lin's and Resnik's definitions. The measure *sim*_*Rel *_takes into account how close terms are to their LCA as well as how detailed the LCA is, i.e., distinguishes between generic and specific terms. This *sim*_*Rel *_score is the basis for a new measure, called *funSim*, for assessing the functional relationship between two gene products. *funSim *extends the measure of similarity to the comparison of two functional annotations, each composed of sets of GO terms from different ontologies. The *funSim *score allows for identifying functionally related gene products from different species that have no significant sequence similarity. The measure also allows for partial matches, resulting in a more robust similarity score for the comparison of gene products with incomplete annotation or for the comparison of multi-functional proteins. We used *sim*_*Rel *_to identify all biological processes from fungi that do not appear in mammals. Furthermore, *sim*_*Rel *_was used to find molecular functions from *Mycobacteria *that do not appear in mammals. We compared the *funSim *score to established sequence similarity approaches. The method was also applied to find the proteins from human that are functionally related to yeast proteins. We compared the yeast proteins with each other using *funSim*, and obtained a functional map using multidimensional scaling. We also applied *funSim *to the functional comparison of all Pfam families and generated a functional map of the protein families.

## Results and discussion

### Comparing biological processes and molecular functions

The *sim*_*Rel *_measure was used to investigate the similarities and differences of the molecular biology between different taxonomic groups. The *sim*_*Rel *_score ranges between 0 and 1. GO terms with a *sim*_*Rel *_score above 0.9 correspond to highly similar functions. Between 0.5 and 0.7, the two GO terms may be considered functionally related and below 0.3, they are not functionally similar. The relationship between *sim*_*Rel *_score and functional similarity is illustrated with some examples. Comparing the GO term "biotin biosynthesis" (GO:0009102) with itself results in a *sim*_*Rel *_score of 0.99993. The score is smaller than 1.0 because *sim*_*Rel *_relies on the probability of the term (see Methods for details). The terms "ATP-dependent chromatin remodeling" (GO:0043044) and "chromatin silencing at telomere" (GO:0006348) have a similarity score of 0.75098. These two terms are both descendants of "chromatin remodelling" (GO:0006338) and represent related biological processes. The biological process "aromatic amino acid transport" (GO:0015801) and "L-glutamate transport" (GO:0015813) have a score of 0.55565. The lowest common ancestor of the two terms, "amino acid transport" (GO:0006865), is rather generic, resulting a low *sim*_*Rel *_score. The process "chitin localization" (GO:0006033) and the unrelated process "ATP synthesis coupled proton transport" (GO:0015986) have a low similarity score (0.30027).

The *sim*_*Rel *_measure was used to find processes from fungi that are not present in mammals. This kind of investigation is of medical interest, as proteins involved in biological processes unique to pathogens and absent in the host are potential drug targets. The fifty most dissimilar biological processes from fungi and mammals are provided in the supplementary material (see Additional file 1, Table S1). "Plasmid partitioning" (GO:0030541) and "chitin localization" (GO:0006033) have the lowest *sim*_*Rel *_scores, 0.15808 and 0.30027 respectively. They are unique to fungi, in particular "chitin localization" is a promising candidate for finding new drug targets [26]. The next step should be to assess the relevance of the individual proteins associated with the selected processes for the survival of the organism. Both "Boron transport" (GO:0046713) and "snoRNA transcription" (GO:0009302) have a low score, which reveals how the comparison results depend on the quality of the functional annotations. The human protein with the UniProt accession Q8NBS3 is actually involved in "boron transport" [27] but this is not yet annotated with GO terms in UniProt. One yeast protein (UniProt accession: P53538) is annotated with "snoRNA transcription" [28]. There is a predicted human orthologous gene in Ensembl (ENSG00000160075) that belongs to the same InterPro family [29] (IPR006811) as the yeast protein, but the human gene product is also not yet annotated with GO.

Additionally, the *sim*_*Rel *_score was used to find molecular functions from the genus *Mycobacterium *that cannot be found in mammals. Our database contains annotations for proteins of several *Mycobacterium *pathogens. *M. avium paratuberculosis *is the causative agent for Johne's disease in ruminants and it is possibly linked to Crohn's disease in humans. *M. bovis *causes tuberculosis in most animals and in cattle in particular. *M. tuberculosis *and *M. leprae *cause tuberculosis and leprosy in humans, respectively. A list of the 60 most dissimilar functions according to *sim*_*Rel *_is given in the Supplement (see Additional file 1, Table S2). The molecular function with the lowest *sim*_*Rel *_score (0.05293) corresponds to "3,4-dihydroxy-2-butanone-4-phosphate synthase activity" (GO:0008686), indicating a molecular function in *Mycobacteria *that is absent in mammals. In fact, this catalytic activity corresponds to one of the first steps in riboflavin biosynthesis. Riboflavin is the precursor of flavocoenzymes which are essential for the catalysis of a variety of redox-reactions. Riboflavin is produced in microorganisms, fungi, and plants but is an essential nutrient for animals. The riboflavin biosynthetic pathway has been considered a potential drug target for anti-infectives against pathogenic fungi, bacteria, and mycobacteria in particular [30,31]. There has also been some specific interest on developing inhibitors of the 3,4-dihydroxy-2-butanone-4-phosphate synthase from different fungi [32,33] but so far there has been no specific study on mycobacteria. Other molecular functions not found in mammals of interest for drug discovery can be found in the list. For example, "UDP-N-acetylmuramate dehydrogenase activity" (GO:0008762), *sim*_*Rel *_= 0.59661, is one step in the synthesis of bacterial peptidoglycan, or "adenosylmethionine-8-amino-7-oxononanoate transaminase activity" (GO:0004015), *sim*_*Rel *_= 0.6486, which is part of the biotin synthesis.

### Comparison of *funSim *and sequence similarity

**Figure 1:**
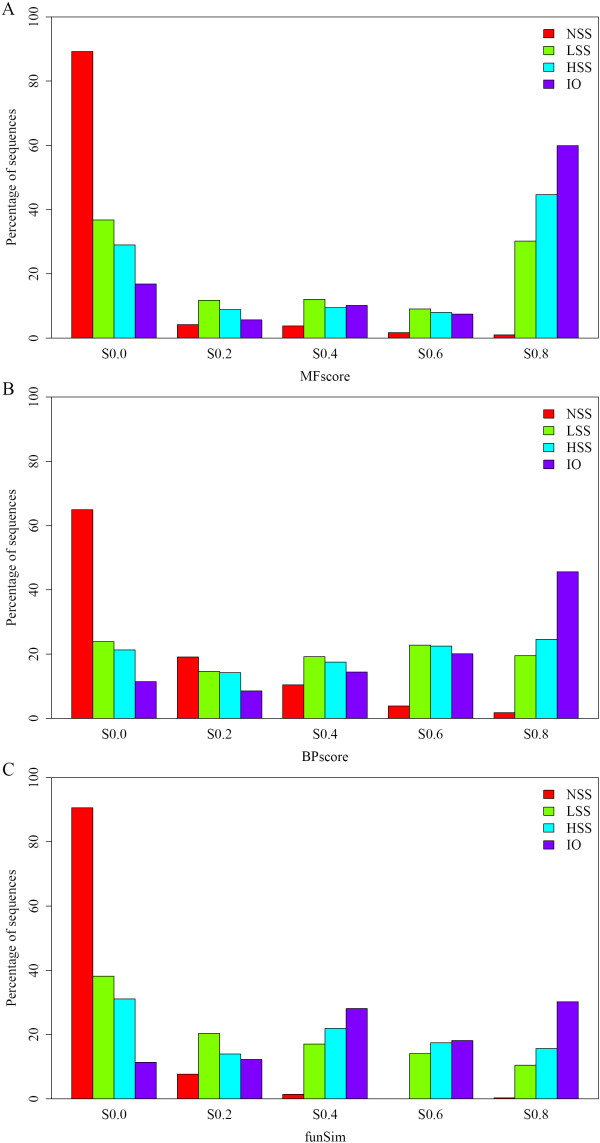
**Distribution of the *MFscore *(A), *BPscore *(B), *funSim *score (C) for different sets of protein pairs excluding IEA and ISS annotation**. The bins correspond to the following intervals of *funSim *values: S0.0: [0.0, 0.2[; S0.2: [0.2, 0.4[; S0.4: [0.4, 0.6[; S0.6: [0.6, 0.8[; S0.8: [0.8, 1.0]. The percentage values are calculated according to the total number of protein pairs in the different categories. The sets of the different categories contain the following numbers of protein pairs: NSS 288, LSS 364, HSS 338, and IO 563. The distributions were calculated by excluding all proteins with GO annotations with the evidence code IEA (inferred from electronic annotation) and ISS (inferred from sequence or structural similarity) from the datasets.

The *funSim *score ranges from 0 to 1, which translates into an increasing degree of functional similarity, in a comparable way to the *sim*_*Rel *_score. This is expected as the *funSim *score is a combination of *sim*_*Rel *_scores. A *funSim *score close to one indicates high functional similarity whereas a score close to zero indicates low similarity. We analyzed the distribution of the *funSim *score and its two components, the *MFscore *(for molecular function) and the *BPscore *(for biological process), in four different categories of protein pairs corresponding to four levels of evolutionary relationship: no sequence similarity (NSS), low sequence similarity (LSS), high sequence similarity (HSS), and orthology according to Inparanoid (IO) [34]. GO annotation with IEA (inferred from electronic annotation) and ISS (inferred from sequence or structural similarity) evidence codes was disregarded. Figures 1A and 1B show the distribution of the *MFscore *and the *BPscore *in the four datasets. Almost 60% of the protein pairs in the IO dataset have an *MFscore *above 0.8 and 45% have a *BPscore *in the same range. This indicates that Inparanoid ortholog proteins tend to have similar molecular functions and are also involved in similar biological processes, although to a smaller extent. Some protein pairs in the IO set have scores below 0.2, indicating no functional similarity. It can be seen in all four datasets (NSS, LSS, HSS, IO), that there are more protein pairs with an intermediate *BPscore *between 0.2 and 0.8 than with a *MFscore *in the same range. This is caused by the lower density of the molecular function ontology. High-level terms in this ontology are less connected than high-level terms in the biological process ontology which results in lower scores for molecular function. The percentage of proteins with high functional similarity (S0.8) is highest for the IO category, and decreases for HSS and LSS, to almost no protein pairs without sequence similarity (NSS). The reverse order is observed for the proteins without functional similarity (S0.0) where the highest percentage is observed for NSS and then in decreasing order LSS, HSS, and IO. This effect is more pronounced for the *MFscore *than for the *BPscore*.

**Figure 2:**
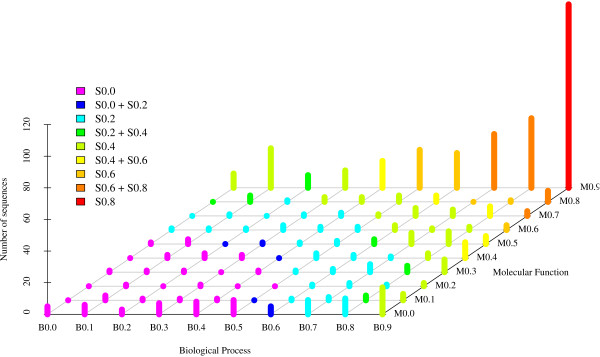
**Distribution of *MFscore *and *BPscore *values for the IO dataset**. The bins correspond to the same intervals as in Figure 1. The bars are colored according to the *funSim *score of the protein pairs contained.

Figure 1C shows the distribution of the *funSim *score for the different datasets. Since the *funSim *score is based on the other two scores, it has an intermediate distribution. About half of the orthologous protein pairs have a score above 0.6 indicating some functional relationship between the proteins. In particular the highest peak is at S0.8 which indicates high functional relatedness of the proteins. Nevertheless, 25% of the orthologous protein pairs have a *funSim *value below 0.4 indicating a very low functional similarity. The IO distribution shows a local peak at S0.4 which is a result of the combination of the *MFscore *and the *BPscore *for *funSim*. A considerable number of protein pairs have a high *MFscore *and a low *BPscore *or vice versa, resulting in *funSim *scores in the range between 0.4 and 0.6, as explained later in Figure 2. The protein pairs in the set NSS have very low scores with few exceptions. This indicates that there is almost no functional relationship between random pairs. The distributions for the LSS and the HSS sets show considerable similarity. However, there is shift in the LSS distribution towards lower scores if compared to the HSS distribution. Figure S1 (see Additional file 1) shows the same type of results as Figure 1 but including all available annotation. There is no considerable difference between the distributions in Figure 1 and Figure S1 (see Additional file 1). The only exception is the distribution of LSS and HSS protein pairs which have a higher percentage of high *BPscores *(S0.8). This is also refected by the *funSim *score, though to a lower extend. In general, excluding the electronic annotations does not have a great effect on the distribution of the similarity scores.

Figure 2 shows a histogram of the relationship between *MFscore *and *BPscore *for the proteins in the IO dataset. The bars are colored according to the *funSim *score of the protein pairs. The highest peak occurs at M0.9 and B0.9, which indicates that many Inparanoid orthologous pairs perform the same function and are involved in the same processes. A considerable number of protein pairs have a high score (higher than 0.8) in one of the ontologies and a low score (lower than 0.2) in the other ontology. This corresponds to the upper left and the lower right corners of the plot. These proteins have either similar molecular function but take part in different biological processes or belong to similar biological processes and perform different molecular functions. These proteins have a *funSim *score between 0.4 and 0.6, resulting in the local peak for ortholog proteins at S0.4 in Figure 1C.

**Figure 3:**
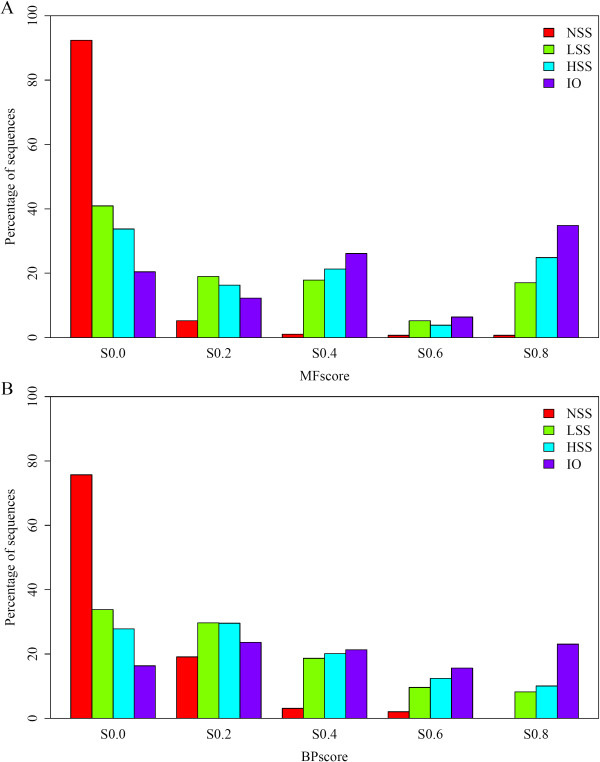
**Distribution of the *MFscore*_*Lord *_(A) and *BPscore*_*Lord *_(B) for different sets of protein pairs excluding IEA and ISS annotation**. The bins correspond to the same intervals as in Figure 1. The percentage values are calculated according to the total number of protein pairs in the different categories. The sets of the different categories contain the following numbers of protein pairs: NSS 288, LSS 364, HSS 338, and IO 563. The distributions were calculated by excluding all proteins with GO annotations with the evidence code IEA (inferred from electronic annotation) and ISS (inferred from sequence or structural similarity) from the datasets.

We compared our measure of functional similarity between gene products to the approach previously proposed by Lord *et al*. [16]. In performing this comparison, we were faced with several challenges; the lack of objective validation sets, the fact that Lord's measure can be arbitrarily large, and the fact that there is no established cutoff for significant similarity for functional similarity measures. However, a partial comparison of the two approaches is still possible regarding the combination of semantic similarity scores. We compared the proposed *MFscore *and *BPscore *to the corresponding *MFscore*_*Lord *_and *BPscore*_*Lord*_, which rely on the average semantic similarity between the GO terms as proposed by Lord (see Methods). In order to obtain scores that range within predefined intervals with Lord's measure, we used *sim*_*Rel *_to estimate the semantic similarity between GO terms. We calculated *MFscore*_*Lord *_and *BPscore*_*Lord*_distributions for the NSS, LSS, HSS, and IO sets. It is expected that most protein pairs in the NSS set are not functionally related and therefore should obtain low *GOscores *whereas pairs in the IO set generally have similar functions. However, the NSS set also contains functionally related proteins that share no significant sequence similarity. Although this prevents an objective performance assessment, the comparison of the shapes of the distributions of the *GOscores *for the NSS and the IO sets provides an indication of the discriminative power of the two approaches. We observe that the shapes of the distributions of *MFscore*_*Lord *_and *BPscore*_*Lord *_(Figure 3) differ from that of the corresponding distributions of *MFscore *and *BPscore *(Figure 1). There is a substantially lower percentage of protein pairs with *MFscore*_*Lord *_above 0.8 than with *MFscore *but a higher percentage of pairs with similarity between 0.2 and 0.6. The *MFscore*_*Lord *_distribution of the IO set has two peaks, one at S0.4 and one at S0.8. Therefore, *MFscore*_*Lord *_does not discriminate as clearly between non-homologous and homologous, and in particular orthologous, proteins as *MFscore *does. The NSS results for *MFscore*_*Lord *_closely resemble the results with *MFscore*. In case of the *BPscore*_*Lord*_, the IO, HSS, and LSS distributions are more uniform without pronounced peaks compared to the *BPscore*. The NSS distribution is again very similar to the distribution obtained with *BPscore*. We performed a *χ*^2^-test to investigate whether the distributions obtained by *MFscore *and *BPscore *differ significantly from the distributions generated by *MFscore*_*Lord *_and *BPscore*_*Lord*_, respectively. Except for the NSS distributions, the *χ*^2^-test supports this expectation with p-values less than 10^-4^.

In summary, these results confirm that functionally related proteins tend to have higher sequence similarity. This is more evident for the *MFscore*. Nevertheless, a considerable percentage of protein pairs that are orthologous and that have a high sequence similarity show no functional similarity. The comparison with Lord's approach to combine semantic similarity scores shows significantly different results. In particular, the proposed approach is expected to provide a better discrimination between non-homologous and orthologous proteins.

### Finding functionally related proteins

**Figure 4:**
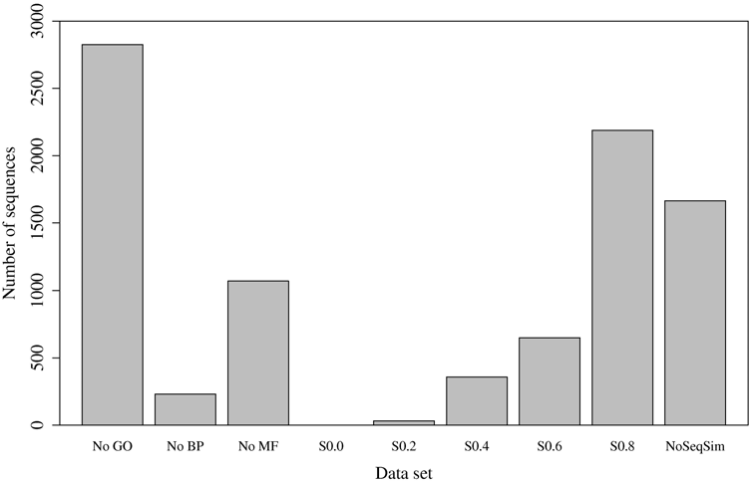
**Functional comparison of yeast proteins with human proteins**. Only the best hit (highest *funSim *score) for each yeast protein was taken into account for the score distribution. For the NoSeqSim bin, a BLAST comparison was performed with default parameters for the proteins with *funSim *above 0.8 (in bin S0.8), and the pairs with no significant sequence similarity (e-value > 10^-3^) were considered.

For each yeast protein, the *funSim *score was used to search for the functionally related proteins in human. As a result of this directional comparison, each yeast protein is mapped to a list of functionally related human proteins sorted by *funSim*. In total, we compared the 7 356 yeast proteins from UniProt to the 70447 proteins from human in UniProt. Figure 4 shows the overall distribution of the highest *funSim *score for each yeast protein. The distribution shows that there are only about 30 yeast proteins with a score below 0.4, which indicates that there is no functionally related protein in human. For almost 2 200 (30%) yeast proteins, there is a functionally very similar protein in human with a score above 0.8. Out of these protein pairs with *funSim *score above 0.8, more than 1 600 have no significant sequence similarity with human proteins (NoSeqSim) and almost 1 400 share no Pfam [35] families with human proteins. These functionally related protein pairs are either non-homologous and evolved independently to a similar function or are remote homologs that cannot be identified by standard sequence-based methods.

We further analyzed some of the yeast-human protein pairs associated with different ranges of *funSim *values. The Glutaredoxin-1 from yeast (UniProt accession: P25373) matches two proteins from human (UniProt accessions: Q6NXQ3, Q5T501) with a very high *funSim *score (0.99968). All these three proteins have glutathione peroxidase activity as response to oxidative stress. According to both SCOP [36] and Pfam, the human proteins are classified in the same family, but the yeast protein belongs to a different family. All three proteins are in the same SCOP superfamily (thioredoxin-like), although there is no significant sequence similarity between the human proteins and the yeast protein.

The phosphoacetylglucosamine mutase from yeast (UniProt accession: P38628) matches one human protein with a considerable *funSim *score of 0.843. This human protein is also a phosphoacetylglucosamine mutase (UniProt accession: O95394) and performs exactly the same function on the same pathway, but the human protein is annotated to a more generic biological process GO term. The two proteins are reported as orthologs by Inparanoid [34]. They have a sequence identity of almost 46% and share two Pfam families. These two proteins are functionally very similar.

Decarboxylating sterol-4-alpha-carboxylate 3-dehydrogenase (UniProt accession: P53199) from yeast is annotated with the molecular function "C-3 sterol dehydrogenase (C-4 sterol decarboxylase) activity" (GO:0000252) and with "ergosterol biosynthesis" (GO:0006696) biological process. The functionally most similar human protein is the sigma 1 isoform 1 variant Opioid receptor (UniProt accessions: Q53GN2, Q5T1J1) with a *funSim *score of 0.5005. It is annotated to the molecular function "C-8 sterol isomerase activity" (GO:0000247) and is involved in the same process as the yeast protein. The two proteins perform different functions but take part in the same processes, which translates into a low *MFscore *(0.0303) and a high *BPscore *(1.0).

The serine/threonine-protein kinase ATG1 (UniProt accession: P53104) from yeast is involved in the "autophagy" (GO:0006914) process. The human protein with the highest *funSim *score (0.507) is phosphorylase b kinase gamma catalytic chain (UniProt accession: P15735), also with serine/threonine protein kinase molecular function according to the GO annotation. However, the human protein is involved in the "glycogen metabolism" (GO:0005977) process. Both proteins share the protein kinase domain from Pfam (Pfam accession: PF00069) and have a sequence similarity of 27%. The proteins have the same molecular function (*MFscore *0.994), but take part in different processes (*BPscore *0.159), the type of functional relationship that tends to be predicted by homology-based methods.

The best hit for the nicotinamide riboside kinase 1 from yeast (UniProt accession: P53915) is the UMP-CMP kinase (UniProt accession: P30085) with a *funSim = *0.303. The yeast protein catalyzes the synthesis of nicotinamide nucleotide from nicotinamide riboside, whereas the human protein catalyzes phosphoryl transfer from ATP to UMP and CMP. The two functions are not related, which is reflected in the low score.

### Yeast-yeast comparison

**Figure 5:**
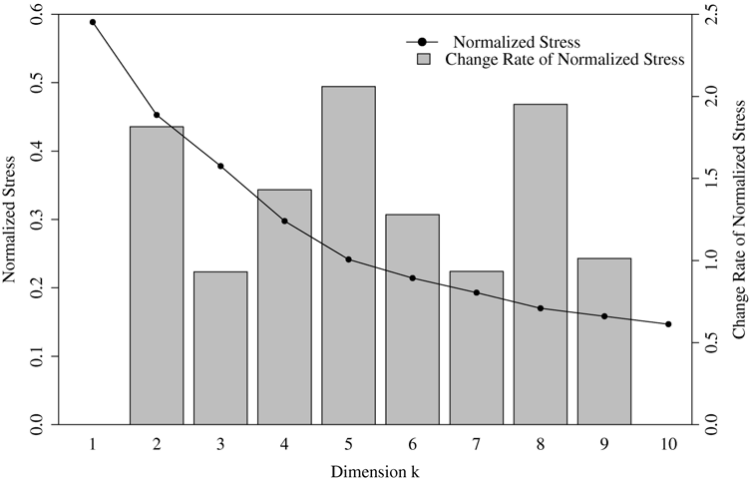
**Scree-plot of multidimensional scaling**. The change rate indicates that a five-dimensional space would be optimal for representing the data. Furthermore, it indicates that using a three dimensional representation does not improve much over a two dimensional representation.

**Figure 6:**
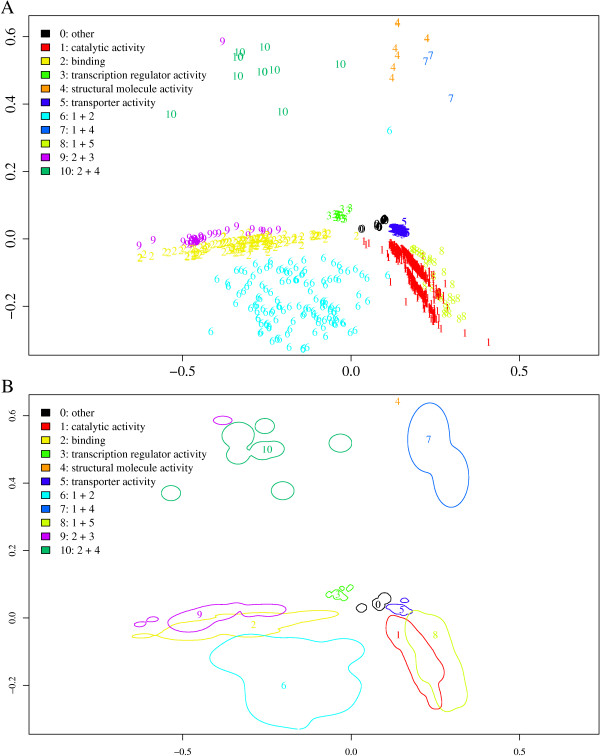
**Yeast functional map**. A: The yeast functional map, obtained by 2D-Multidimensional scaling of an all-against-all comparison of yeast proteins using *d*_*mf*_. The proteins are represented by numbers in the plot and are colored according to their type of molecular function. The plot shows that the proteins group into clusters according to their different functions. Additionally, proteins annotated with two terms are placed between the clusters that correspond to the single functions. B: Contour plot of the MDS.

Based on the *MFscore*, the *d*_*mf *_score was defined as a measure for functional distance with regard to the molecular function (see Methods section). This score is calculated as *d*_*mf *_= 1 - *MFscore*. We computed *d*_*mf *_scores for all pairwise combinations of yeast proteins. The underlying dataset consists of all yeast proteins from UniProt with molecular function annotation, 3 459 proteins in total, resulting in 5980611 unique protein pairs. Approximately 5.3 million pairwise distances were larger than 0.8, indicating no functional similarity. Slightly more than 104 000 protein pairs had a distance below 0.2, suggesting high functional similarity. The *d*_*mf *_scores have been used as input for metric multidimensional scaling (MDS) and clustering in order to group the proteins according to their function. Previously, proteins have been grouped according to sequence or structure in a similar way [37-39]. Generally, the goal of MDS is to represent points from a high dimensional space in a lower dimensional space while preserving the pairwise distances of the term. Normalized stress is a measure of how well the pairwise distances are preserved in the lower dimensional space. Figure 5 shows the plot with the normalized stress (NS) and the change rate of normalized stress (CR). NS is a measure of how well the original distances are represented in the dataset with reduced dimensionality. The highest CR indicates the optimal number of dimensions to represent the original dataset. The normalized stress for the two-dimensional (2D) MDS of the dataset is 0.45, and the plot indicates that there is not much improvement in NS by using three dimensions instead of using two dimensions. The 2D MDS of the dataset corresponds to the map of the yeast functional space, and is shown in Figure 6A. The contour plot in Figure 6B shows the regions corresponding to different functions. Different colors were chosen to match certain high-level terms that are children of "molecular_function" and for some combinations of these high-level terms. Proteins annotated with "catalytic activity" (1) are arranged along lines in the lower right part of the plot. Proteins with "binding" (2) annotation are located on an axis, approximately parallel to the x-axis to the left of the origin. Proteins annotated with both of these classes (6) are placed between these two clusters. In general, proteins with the same function form clusters along axes and proteins annotated with two different functions are placed between the corresponding clusters. Overall, the yeast proteins with different types of molecular functions are well separated in the MDS plot.

**Figure 7:**
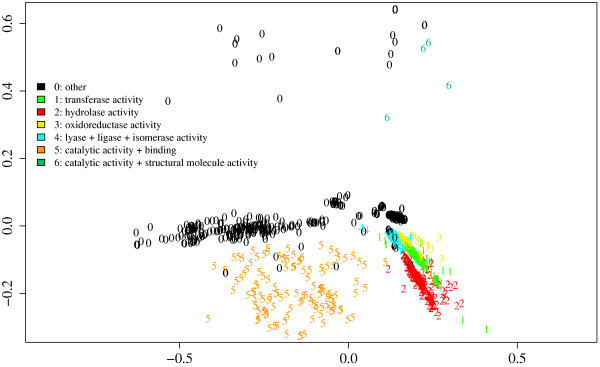
**2D-Multidimensional scaling plot colored according to the type of "catalytic activity" of the proteins**. Proteins annotated with a descendant are colored according to the type of their catalytic activity. One elongated region corresponds to "transferase activity" (1), another to "hydrolase activity" (2), and another region to "oxidoreductase activity" (3). Proteins annotated with "lyase activity", "ligase activity", or "isomerase activity" (4) are mostly located along the top of the whole "catalytic activity" region.

**Figure 8:**
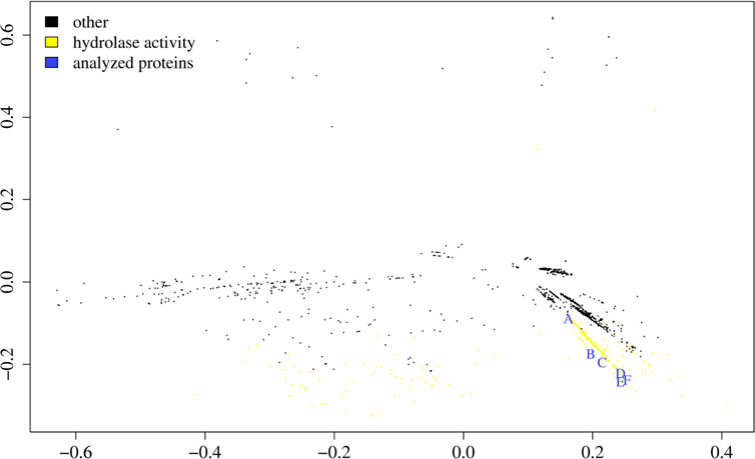
**Detailed analysis of "hydrolase activity"**. Proteins annotated with a descendant of "hydrolase activity" are shown in yellow. The six marked proteins (A to F) are all annotated with a single molecular function. The proteins are annotated with the GO terms as follows: Protein A (YBR177C), "serine hydrolase activity" (*p *= 5.277 * 10^-6^); Protein B (DBP7), "ATP-dependent RNA helicase activity" (*p *= 4.22 * 10^-5^); Protein C (YAL048C), "GTPase activity" (*p *= 8.69 * 10^-4^); Protein D (Q36760), "endonuclease activity" (*p *= 8.96 * 10^-3^); Protein E (YDL100C), "ATPase activity" (*p *= 2.24 * 10^-2^); Protein F (IAH1), "hydrolase activity, acting on ester bonds" (*p *= 2.71 * 10^-2^). The probability of the annotated term to occur increases moving on the line from A to F. This shows that proteins annotated with more general terms have a larger distance to all other proteins and thus are placed towards the edges of the plot.

We further investigated how well the *MFscore *discriminates between proteins with different types of "catalytic activity". Different colors were chosen to match a subset of children of "catalytic activity" (Figure 7). It becomes evident that different regions correspond to different functional subtypes. The arrangement of common functional subtypes was analyzed in further detail by selecting six proteins annotated with a molecular function term descendant of "hydrolase activity" (Figure 8). In general, the probability of occurrence of the annotated term rises from the center to the edges of the plot. This means that proteins located farther away from the origin are annotated with more generic and therefore less relevant GO terms. The same analysis with the *BPscore *showed no clear separation of the different processes. This is possibly due to the increased density (connectivity) of the biological process ontology in comparison to the molecular function ontology.

**Figure 9:**
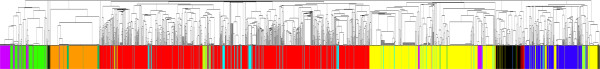
**Hierarchical clustering of all yeast proteins using distance based on the MFscore**. The color bar below the dendogram uses the same color scheme as Figure 6A to indicate the molecular function annotation of the proteins. The dendogram closely resembles the MDS of the yeast proteins. Five clusters can be seen in the dendogram: "catalytic activity" in red, "binding" in pink, "transcription regulator activity" in light green, "structural molecule activity" in orange, and "transporter activity" in dark blue. The dendogram was produced with the JavaTreeView software .

The same distance matrix was used to perform a hierarchical clustering of all yeast proteins according to their molecular function annotation. Figure 9 shows the resulting dendrogram. The colors were chosen to match the categories in the MDS plot (see Figure 6A). It can be seen that the five high-level functions form distinct clusters. The largest cluster "catalytic activity" is plotted in red. This cluster also contains proteins annotated with additional terms (labels 6 and 8 in Figure 6A). Proteins annotated with two different functional classes are placed into either one of the corresponding clusters. Generally, clustering with *d*_*mf *_separates the yeast proteins according to their function, but the separation is not as clear as with multidimensional scaling.

### Applying *funSim *to Pfam families

**Figure 10:**
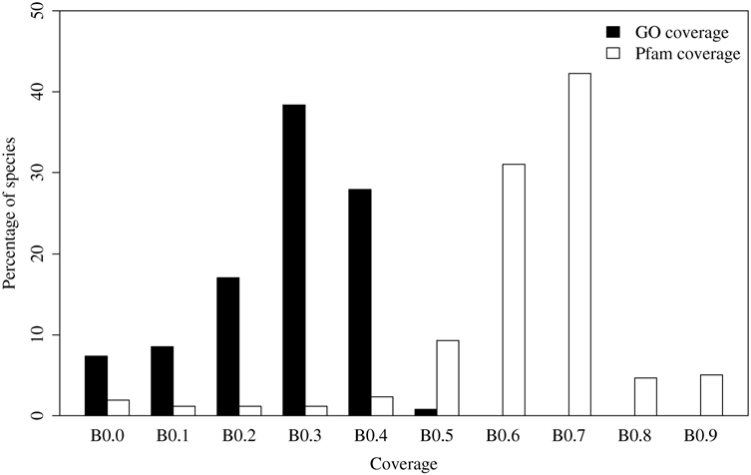
**Distribution of the GO coverage and Pfam coverage**. Distribution of the GO coverage and Pfam coverage from UniProt proteins of completely sequenced genomes. GO coverage means proteins annotated with molecular function and biological process and disregarding cellular component. The mean GO coverage for species in the database is 32%. The Pfam annotation is more complete with a mean of 67%. The bins correspond to the following intervals of coverage: B0.0: [0.0, 0.1[; B0.1: [0.1, 0.2[; B0.2: [0.2, 0.3[; B0.3: [0.3, 0.4[; B0.4: [0.4, 0.5[; B0.5: [0.5, 0.6[; B0.6: [0.6, 0.7[; B0.7: [0.7, 0.8[; B0.8: [0.8, 0.9[; B0.9: [0.9, 1.0].

**Figure 11:**
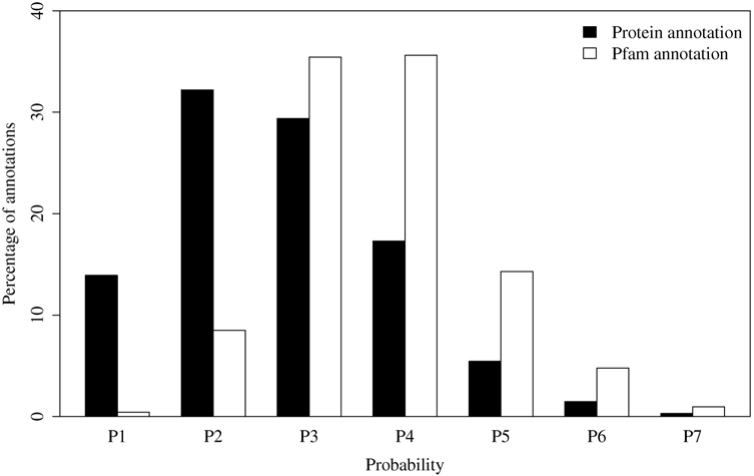
**Distribution of probability values for GO terms annotated to human proteins or human Pfams**. The bins correspond to the following intervals of GO term probability: P1: [0.0, 10^-7^[; P2: [10^-7^, 10^-6^[; P3: [10^-6^, 10^-5^[; P4: [10^-5^, 10^-4^[; P5: [10^-4^, 10^-3^[; P6: [10^-3^, 10^-2^[; P7: [10^-2^, 10^-1^[.

Protein families can also be compared with the *funSim *measure, since most Pfam families are also annotated with GO terms. A *funSim *comparison based on Pfam families is actually preferred for the genomes for which the coverage of the GO annotation of the gene products is rather low, but with a rather high Pfam annotation coverage. In general for the completely sequenced genomes, the Pfam coverage is higher than the GO coverage (Figure 10). One drawback of the family-based functional comparison is that the Pfam families are generally annotated with more generic terms than gene products, because the functional annotation of a family has to fit all its member proteins. The higher the probability of a GO term, the more generic it is. Comparing the probabilities of GO annotations of human proteins and the probabilities of GO annotation of human protein families, it is clear that the Pfam annotation is more generic than the annotation of the gene products (Figure 11). However, this is not always the case. Some genomes have been annotated mostly using automated procedures based on sequence similarity, including Pfam searches with Hidden Markov Models. In such cases, the gene product annotation will correspond to the functions shared by the different family members and therefore will match more closely the Pfam annotation.

**Figure 12:**
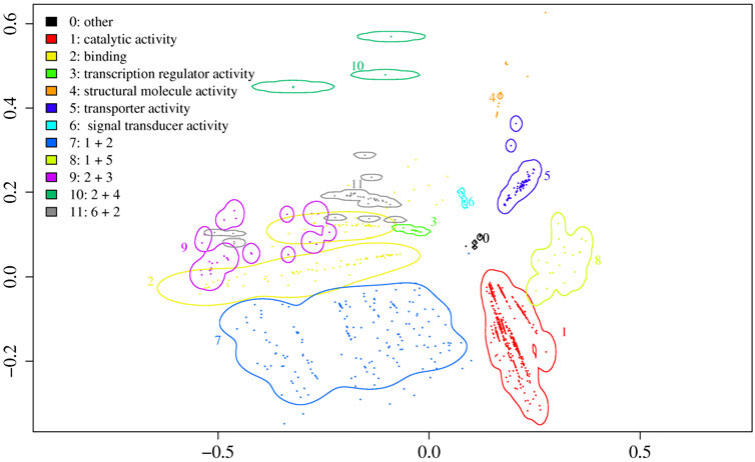
**Functional map of the Pfam families**. Plot of the two-dimensional multidimensional scaling of the Pfam families. The colors were chosen to resemble the molecular function annotation.

**Figure 13:**
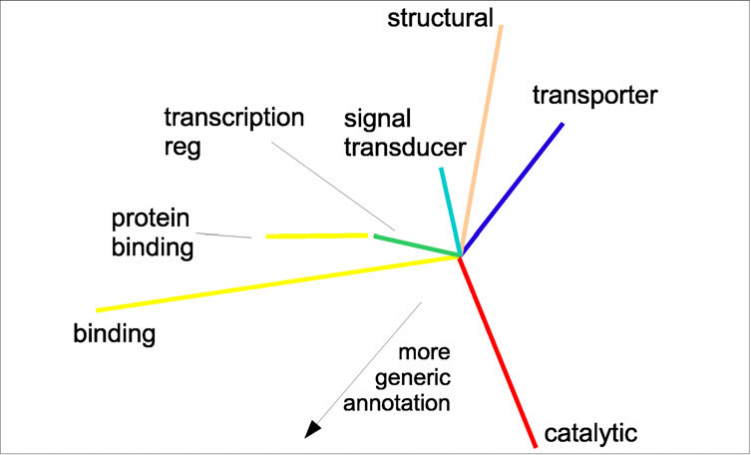
**Axes on the Pfam functional map**. The main axes of the main clusters from Figure 12 are shown.

Using the *d*_*mf *_score, we calculated all possible pairwise functional differences for all Pfam families with molecular function annotation. The resulting distance matrix was used to perform a 2D MDS, in order to obtain a map of the Pfam functional space. Figure 12 shows the graphical representation of the 2D MDS. The protein families are colored according to their molecular function annotation. It can be seen that Pfams with the same function form rather well defined clusters. Overlapping clusters always contain families that are annotated with one common and possibly one additional function. Protein families in some clusters are arranged along axes where families annotated to more general GO terms locate towards the edges of the plot. Regions of constant density are shown as contour lines in the plot. They reveal a quite substantial overlap of the clusters 2 and 9 which both contain Pfams annotated to "binding". Additionally, cluster 2 is split into two distinct regions that are quite large. An analysis of the two cluster parts shows that the upper part contains Pfams annotated to "protein binding" (GO:0005515) and the lower part contains Pfams annotated with other kinds of "binding". Figure 13 shows the different axes of the main clusters in the map of the Pfam functional space.

## Conclusion

As a result of the genome annotation process, an increasing amount of functional information is being accumulated in a systematic and machine-readable fashion. This affords a computational approach to comparing gene products based on their functional annotation. Such a strategy bears the promise of a more direct functional comparison than traditional sequence comparison methods. The new approach is not intended to be a replacement of the sequence comparison or homology-based approaches but rather provides an additional alternative for the objective comparison of the annotated gene products. Here we propose two new measures for the comparison and identification of functionally related gene products. The *sim*_*Rel *_score provides a similarity measure of two GO terms. It combines the power of Resnik's and Lin's measures in the sense that both the relevance of the LCA and the distance to the LCA are taken into account. The *funSim *score is based on *sim*_*Rel *_and compares the GO annotation of two gene products. The score compares sets of GO terms from different ontologies, and it allows for partial matches. Additionally, the *d*_*mf *_score is based on the *MFscore *and is used to measure functional distances. Similar distance measures can be definied for the *sim*_*Rel *_score, the *BPscore*, and the *funSim *score.

The *MFscore*, *BPscore*, and the *funSim *score allow for partial matches, therefore they are suitable for the comparison of multi-functional gene products. In addition, these measures are also suitable for the comparison of gene products for which only part of the functional annotation is available as GO terms. This can be illustrated by the previous comparison of Glutaredoxin-1 from yeast (P25373) and the GPX3 protein from human (Q6NXQ3). The yeast and human proteins share a peroxidase activity, but the yeast protein is also annotated as a transferase. The proteins clearly share similar function, which is refected by the high *funSim *score (0.99968), although the yeast protein is annotated with additional functions. Nevertheless, such sequence-independent similarity measures are always limited by the availability and quality of the functional annotations and their underlying ontologies. This is refected by the previously mentioned missing "boron transport" annotation for the human protein Q8NBS3, making it impossible to find functionally related proteins in yeast.

Other measures have been proposed for functional comparison of gene products (see Introduction). They are based either on Resnik's or Lin's similarity measures. Therefore, they do not consider both the distance to the LCA and the relevance of the LCA. In addition, these measures do not explicitly take into account partial matches, as they penalize all mismatches or consider only the best single match. The comparison of our measures with Lord's approach [16] is limited by the lack of a gold standard for either true positives or true negatives. Therefore, one is restricted to the comparison of the shapes of the distributions of scores. If Lord's approach to combining semantic similarity scores is used, the results differ significantly from the ones obtained with the current approach. The latter approach provides a better discrimination between non-homologous and homologous, particularly orthologous proteins. Future progress in this area requires an objective criterion for testing the performance of the different measures of functional similarity.

There are several general application scenarios for the proposed measures. The *sim*_*Rel *_score is used to compare two sets of GO terms in order to find functional terms that are common to both sets and unique to each set, respectively. This is especially valuable for the comparison of the underlying molecular biology of different groups of organisms along the taxonomic tree. The comparison of the biological processes from fungi and mammals given in the Results section is one such example. Additionally, the *sim*_*Rel *_score could be applied in the characterization of the functional diversity of organism communities in different environments [40]. In the second application scenario gene products are compared using the *funSim *score in order to find functional relationships. All gene products from a single genome are compared and grouped according to function. An example is the multidimensional scaling and the cluster analysis of the yeast proteins (see Figures 6A, 9). A similar analysis can be performed on protein families in order to generate a map of the family functional space. Alternatively, two genomes are compared to find functionally similar gene products and to identify gene products unique to one of the species, respectively, as in the comparison between yeast and human proteins (see Figure 4). To summarize, the approach enables the comparison of the molecular functions and biological processes found in different groups of organisms and provides a new tool to identify functionally related gene products independent of homology.

One can foresee applications that are not only biologically but also medically relevant. In particular, these comparisons can provide better understanding of pathogenicity and aid in the identification of new drug targets. For example, established comparative genomics approaches for drug target discovery are based on sequence similarity searches [41,42], and can be extended to include semantic similarity searches for functional comparison.

Although this approach is promising, the quality of the results is still quite sensitive to the quality of the annotations. However, there is reason to be optimistic, since the situation is expected to improve as new GO terms are added and as more genes are annotated. The "is-a" and "part-of" relationships between GO terms are not distinguished in the current approach. This problem should be addressed in the future. Another possible extension is to include cellular component into the *funSim *score in order to completely assess the function and the cellular location of a gene product.

A future goal is to identify functionally equivalent gene products from different genomes. They perform the same molecular functions, take part in the same biological processes and are located in the same cellular component. The definition of functional equivalence is more generic than that of orthology as it does not depend on homology. The *funSim *score can be used as a basis for defining a new measure to identify the functionally equivalent gene products from different species.

## Methods

### Database

A database (GOTaxDB) was implemented that integrates information from different sources. The database contains the NCBI Taxonomy [43] downloaded on August 22nd, 2005. Furthermore, we imported Pfam 18.0 [35] released in July 2005 and the SMART domains [44] from the InterPro release 11.0 [29]. The Gene Ontology [10] term definitions were taken from the monthly release from August 2005. The protein information and annotations were imported from UniProt [12] release 5.8 from August 2005. We implemented a program, GOTaxExplorer, to easily execute the queries and to allow searches involving all integrated sources. The program is freely available over the internet at .

### GO term probability

The probability of a term to occur is assumed to be equal to its frequency in the annotations in a database [16]. The frequency of a term is given by

freq(c)=anno(c)+∑h∈children(c)freq(h).     (1)
 MathType@MTEF@5@5@+=feaafiart1ev1aaatCvAUfKttLearuWrP9MDH5MBPbIqV92AaeXatLxBI9gBaebbnrfifHhDYfgasaacH8akY=wiFfYdH8Gipec8Eeeu0xXdbba9frFj0=OqFfea0dXdd9vqai=hGuQ8kuc9pgc9s8qqaq=dirpe0xb9q8qiLsFr0=vr0=vr0dc8meaabaqaciaacaGaaeqabaqabeGadaaakeaacqWGMbGzcqWGYbGCcqWGLbqzcqWGXbqCcqGGOaakcqWGJbWycqGGPaqkcqGH9aqpcqWGHbqycqWGUbGBcqWGUbGBcqWGVbWBcqGGOaakcqWGJbWycqGGPaqkdaaeqbqaaiabdAgaMjabdkhaYjabdwgaLjabdghaXjabcIcaOiabdIgaOjabcMcaPaWcbaGaemiAaGMaeyicI4Saem4yamMaemiAaGMaemyAaKMaemiBaWMaemizaqMaemOCaiNaemyzauMaemOBa4MaeiikaGIaem4yamMaeiykaKcabeqdcqGHris5aOGaeiOla4IaaCzcaiaaxMaadaqadaqaaiabigdaXaGaayjkaiaawMcaaaaa@5EC1@

*anno*(*c*)+ is the number of gene products annotated with this term in the database. *children*(*c*) is the set of child nodes of term *c*. The probability of term *t *is then defined as *p*(*c*) = *freq*(*c*)/*freq*(*root*), where *freq*(*root*) is the frequency of the root term. The probability is calculated independently for each ontology. It is monotonically increasing as one moves up on a path from a leaf to the root.

### Resnik's measure

Resnik uses the concept of "information content" (IC) to define a semantic similarity measure. The information content is based on the probability *p*(*c*) of a term and measures the amount of information. The probability assigned to a term is defined as its relative frequency of occurrence. The root has probability *p*(*root*) = 1 if it is unique. Resnik uses the negative logarithm to the base 10 of the term's probability, *IC*(*c*) = -log_10 _*p*(*c*), as information content. The more information two terms share the higher is their similarity. The shared information is captured by the set of common ancestors in the graph. The amount of shared information and thus the similarity between the two terms is quantified by the information content of the common ancestors. This leads to the following formula for semantic similarity between two terms in an ontology:

simResnik(c1,c2)=max⁡c∈S(c1,c2)(−log⁡p(c)),     (2)
 MathType@MTEF@5@5@+=feaafiart1ev1aaatCvAUfKttLearuWrP9MDH5MBPbIqV92AaeXatLxBI9gBaebbnrfifHhDYfgasaacH8akY=wiFfYdH8Gipec8Eeeu0xXdbba9frFj0=OqFfea0dXdd9vqai=hGuQ8kuc9pgc9s8qqaq=dirpe0xb9q8qiLsFr0=vr0=vr0dc8meaabaqaciaacaGaaeqabaqabeGadaaakeaacqWGZbWCcqWGPbqAcqWGTbqBdaWgaaWcbaacbiGae8NuaiLae8xzauMaem4CamNaemOBa4MaemyAaKMaem4AaSgabeaakiabcIcaOiabdogaJnaaBaaaleaacqaIXaqmaeqaaOGaeiilaWIaem4yam2aaSbaaSqaaiabikdaYaqabaGccqGGPaqkcqGH9aqpdaWfqaqaaiGbc2gaTjabcggaHjabcIha4bWcbaGaem4yamMaeyicI4Saem4uamLaeiikaGIaem4yam2aaSbaaWqaaiabigdaXaqabaWccqGGSaalcqWGJbWydaWgaaadbaGaeGOmaidabeaaliabcMcaPaqabaGccqGGOaakcqGHsislcyGGSbaBcqGGVbWBcqGGNbWzcqWGWbaCcqGGOaakcqWGJbWycqGGPaqkcqGGPaqkcqGGSaalcaWLjaGaaCzcamaabmaabaGaeGOmaidacaGLOaGaayzkaaaaaa@6157@

where *S*(*c*_1_, *c*_2_) is the set of common ancestors of terms *c*_1 _and *c*_2_. The lowest common ancestor (LCA) is the argmaxc∈S(c1,c2)(−log⁡p(c))
 MathType@MTEF@5@5@+=feaafiart1ev1aaatCvAUfKttLearuWrP9MDH5MBPbIqV92AaeXatLxBI9gBaebbnrfifHhDYfgasaacH8akY=wiFfYdH8Gipec8Eeeu0xXdbba9frFj0=OqFfea0dXdd9vqai=hGuQ8kuc9pgc9s8qqaq=dirpe0xb9q8qiLsFr0=vr0=vr0dc8meaabaqaciaacaGaaeqabaqabeGadaaakeaaieGacqWFHbqycqWFYbGCcqWFNbWzcqWFTbqBcqWFHbqycqWF4baEdaWgaaWcbaGaem4yamMaeyicI4Saem4uamLaeiikaGIaem4yam2aaSbaaWqaaiabigdaXaqabaWccqGGSaalcqWGJbWydaWgaaadbaGaeGOmaidabeaaliabcMcaPaqabaGccqGGOaakcqGHsislcyGGSbaBcqGGVbWBcqGGNbWzcqWGWbaCcqGGOaakcqWGJbWycqGGPaqkcqGGPaqkaaa@4BB6@. The minimum similarity is zero and there is no maximum for this measure.

### Lin's measure

Lin defines the similarity between two terms as the ratio of the commonality of the terms and the information needed to fully describe the two terms. The commonality of the terms is again captured by their common ancestors. The information needed to fully describe both terms is the sum of their information, since the random selection of one term is independent of the random selection of the second term. This defining equation is given by

simLin(c1,c2)=max⁡c∈S(c1,c2)(2⋅log⁡p(c)log⁡p(c1)+log⁡p(c2)).     (3)
 MathType@MTEF@5@5@+=feaafiart1ev1aaatCvAUfKttLearuWrP9MDH5MBPbIqV92AaeXatLxBI9gBaebbnrfifHhDYfgasaacH8akY=wiFfYdH8Gipec8Eeeu0xXdbba9frFj0=OqFfea0dXdd9vqai=hGuQ8kuc9pgc9s8qqaq=dirpe0xb9q8qiLsFr0=vr0=vr0dc8meaabaqaciaacaGaaeqabaqabeGadaaakeaacqWGZbWCcqWGPbqAcqWGTbqBdaWgaaWcbaGaemitaWKaemyAaKMaemOBa4gabeaakiabcIcaOiabdogaJnaaBaaaleaacqaIXaqmaeqaaOGaeiilaWIaem4yam2aaSbaaSqaaiabikdaYaqabaGccqGGPaqkcqGH9aqpdaWfqaqaaiGbc2gaTjabcggaHjabcIha4bWcbaGaem4yamMaeyicI4Saem4uamLaeiikaGIaem4yam2aaSbaaWqaaiabigdaXaqabaWccqGGSaalcqWGJbWydaWgaaadbaGaeGOmaidabeaaliabcMcaPaqabaGcdaqadaqaamaalaaabaGaeGOmaiJaeyyXICTagiiBaWMaei4Ba8Maei4zaCMaemiCaaNaeiikaGIaem4yamMaeiykaKcabaGagiiBaWMaei4Ba8Maei4zaCMaemiCaaNaeiikaGIaem4yam2aaSbaaSqaaiabigdaXaqabaGccqGGPaqkcqGHRaWkcyGGSbaBcqGGVbWBcqGGNbWzcqWGWbaCcqGGOaakcqWGJbWydaWgaaWcbaGaeGOmaidabeaakiabcMcaPaaaaiaawIcacaGLPaaacqGGUaGlcaWLjaGaaCzcamaabmaabaGaeG4mamdacaGLOaGaayzkaaaaaa@73A3@

*S*(*c*_1_, *c*_2_) again is the set of common ancestors of terms *c*_1 _and *c*_2_. In contrast to Resnik's similarity, the values range between 0 and 1.

### Relevance similarity

In order to take relevance information into account, we combine Lin's and Resnik's similarity measures. The probability of the LCA reflects its level of detail. Generic terms do not have a high relevance for the comparison of the exact function of different gene products. This results in the definition

simRel(c1,c2)=max⁡c∈S(c1,c2)(2⋅log⁡p(c)log⁡p(c1)+log⁡p(c2)⋅(1−p(c))).     (4)
 MathType@MTEF@5@5@+=feaafiart1ev1aaatCvAUfKttLearuWrP9MDH5MBPbIqV92AaeXatLxBI9gBaebbnrfifHhDYfgasaacH8akY=wiFfYdH8Gipec8Eeeu0xXdbba9frFj0=OqFfea0dXdd9vqai=hGuQ8kuc9pgc9s8qqaq=dirpe0xb9q8qiLsFr0=vr0=vr0dc8meaabaqaciaacaGaaeqabaqabeGadaaakeaacqWGZbWCcqWGPbqAcqWGTbqBdaWgaaWcbaacbiGae8NuaiLae8xzauMaemiBaWgabeaakiabcIcaOiabdogaJnaaBaaaleaacqaIXaqmaeqaaOGaeiilaWIaem4yam2aaSbaaSqaaiabikdaYaqabaGccqGGPaqkcqGH9aqpdaWfqaqaaiGbc2gaTjabcggaHjabcIha4bWcbaGaem4yamMaeyicI4Saem4uamLaeiikaGIaem4yam2aaSbaaWqaaiabigdaXaqabaWccqGGSaalcqWGJbWydaWgaaadbaGaeGOmaidabeaaliabcMcaPaqabaGcdaqadaqaamaalaaabaGaeGOmaiJaeyyXICTagiiBaWMaei4Ba8Maei4zaCMaemiCaaNaeiikaGIaem4yamMaeiykaKcabaGagiiBaWMaei4Ba8Maei4zaCMaemiCaaNaeiikaGIaem4yam2aaSbaaSqaaiabigdaXaqabaGccqGGPaqkcqGHRaWkcyGGSbaBcqGGVbWBcqGGNbWzcqWGWbaCcqGGOaakcqWGJbWydaWgaaWcbaGaeGOmaidabeaakiabcMcaPaaacqGHflY1cqGGOaakcqaIXaqmcqGHsislcqWGWbaCcqGGOaakcqWGJbWycqGGPaqkcqGGPaqkaiaawIcacaGLPaaacqGGUaGlcaWLjaGaaCzcamaabmaabaGaeGinaqdacaGLOaGaayzkaaaaaa@7DEB@

Like *sim*_*Lin*_, *sim*_*Rel *_is symmetric, i.e. *sim*_*Rel*_(*c*_1_, *c*_2_) = *sim*_*Rel*_(*c*_2_, *c*_1_), and also attains values in the interval [0, 1]. Since the relevance of a term decreases with increasing probability, the similarity is weighted with 1 - *p*(*c*) in the computation of *sim*_*Rel*_.

### Calculation of funSim

The first step in the comparison of two gene products is the pairwise comparison of their GO mappings. The mappings to the different ontologies (molecular function and biological process) are examined separately. Considering two gene products A and B annotated with the sets *GO*^*A *^and *GO*^*B *^of GO terms with sizes *N *and *M*, respectively, a similarity matrix *S *is calculated. This matrix contains all pairwise similarity values of mappings GOiA
 MathType@MTEF@5@5@+=feaafiart1ev1aaatCvAUfKttLearuWrP9MDH5MBPbIqV92AaeXatLxBI9gBaebbnrfifHhDYfgasaacH8akY=wiFfYdH8Gipec8Eeeu0xXdbba9frFj0=OqFfea0dXdd9vqai=hGuQ8kuc9pgc9s8qqaq=dirpe0xb9q8qiLsFr0=vr0=vr0dc8meaabaqaciaacaGaaeqabaqabeGadaaakeaacqWGhbWrcqWGpbWtdaqhaaWcbaGaemyAaKgabaGaemyqaeeaaaaa@317D@ of gene product A and mappings GOjB
 MathType@MTEF@5@5@+=feaafiart1ev1aaatCvAUfKttLearuWrP9MDH5MBPbIqV92AaeXatLxBI9gBaebbnrfifHhDYfgasaacH8akY=wiFfYdH8Gipec8Eeeu0xXdbba9frFj0=OqFfea0dXdd9vqai=hGuQ8kuc9pgc9s8qqaq=dirpe0xb9q8qiLsFr0=vr0=vr0dc8meaabaqaciaacaGaaeqabaqabeGadaaakeaacqWGhbWrcqWGpbWtdaqhaaWcbaGaemOAaOgabaGaemOqaieaaaaa@3181@ of gene product B.

*s*_*ij *_= *sim*(GOiA
 MathType@MTEF@5@5@+=feaafiart1ev1aaatCvAUfKttLearuWrP9MDH5MBPbIqV92AaeXatLxBI9gBaebbnrfifHhDYfgasaacH8akY=wiFfYdH8Gipec8Eeeu0xXdbba9frFj0=OqFfea0dXdd9vqai=hGuQ8kuc9pgc9s8qqaq=dirpe0xb9q8qiLsFr0=vr0=vr0dc8meaabaqaciaacaGaaeqabaqabeGadaaakeaacqWGhbWrcqWGpbWtdaqhaaWcbaGaemyAaKgabaGaemyqaeeaaaaa@317D@, GOjB
 MathType@MTEF@5@5@+=feaafiart1ev1aaatCvAUfKttLearuWrP9MDH5MBPbIqV92AaeXatLxBI9gBaebbnrfifHhDYfgasaacH8akY=wiFfYdH8Gipec8Eeeu0xXdbba9frFj0=OqFfea0dXdd9vqai=hGuQ8kuc9pgc9s8qqaq=dirpe0xb9q8qiLsFr0=vr0=vr0dc8meaabaqaciaacaGaaeqabaqabeGadaaakeaacqWGhbWrcqWGpbWtdaqhaaWcbaGaemOAaOgabaGaemOqaieaaaaa@3181@), ∀*i *∈ {1,...,*N*}, ∀*j *∈ {1,...,*M*}     (5)

The matrix may be calculated with any of the similarity measures mentioned above (*sim*_*Resnik*_*, sim*_*Lin*_, and *sim*_*Rel*_). The matrix *S *is not necessarily symmetric or square since the proteins can have different types and numbers of GO mappings. The rows and the columns of *S *represent two different directional comparisons, row vectors correspond to a comparison of A to B and column vectors of B to A. The best hits for the comparison of A with B are determined as maximum values in the rows in matrix *S *(row maxima). The maximum values in the columns of *S *(column maxima) are the best hits for the direction B to A. The averages over the row maxima and the column maxima give similarity values for the comparison of A to B and the comparison of B to A, respectively:

rowScore=1N∑i=1Nmax⁡1≤j≤Msij,     (6)
 MathType@MTEF@5@5@+=feaafiart1ev1aaatCvAUfKttLearuWrP9MDH5MBPbIqV92AaeXatLxBI9gBaebbnrfifHhDYfgasaacH8akY=wiFfYdH8Gipec8Eeeu0xXdbba9frFj0=OqFfea0dXdd9vqai=hGuQ8kuc9pgc9s8qqaq=dirpe0xb9q8qiLsFr0=vr0=vr0dc8meaabaqaciaacaGaaeqabaqabeGadaaakeaacqWGYbGCcqWGVbWBcqWG3bWDcqWGtbWucqWGJbWycqWGVbWBcqWGYbGCcqWGLbqzcqGH9aqpdaWcaaqaaiabigdaXaqaaiabd6eaobaadaaeWbqaamaaxababaGagiyBa0MaeiyyaeMaeiiEaGhaleaacqaIXaqmcqGHKjYOcqWGQbGAcqGHKjYOcqWGnbqtaeqaaaqaaiabdMgaPjabg2da9iabigdaXaqaaiabd6eaobqdcqGHris5aOGaem4Cam3aaSbaaSqaaiabdMgaPjabdQgaQbqabaGccqGGSaalcaWLjaGaaCzcamaabmaabaGaeGOnaydacaGLOaGaayzkaaaaaa@55BB@

columnScore=1M∑j=1Mmax⁡1≤i≤Nsij.     (7)
 MathType@MTEF@5@5@+=feaafiart1ev1aaatCvAUfKttLearuWrP9MDH5MBPbIqV92AaeXatLxBI9gBaebbnrfifHhDYfgasaacH8akY=wiFfYdH8Gipec8Eeeu0xXdbba9frFj0=OqFfea0dXdd9vqai=hGuQ8kuc9pgc9s8qqaq=dirpe0xb9q8qiLsFr0=vr0=vr0dc8meaabaqaciaacaGaaeqabaqabeGadaaakeaacqWGJbWycqWGVbWBcqWGSbaBcqWG1bqDcqWGTbqBcqWGUbGBcqWGtbWucqWGJbWycqWGVbWBcqWGYbGCcqWGLbqzcqGH9aqpdaWcaaqaaiabigdaXaqaaiabd2eanbaadaaeWbqaamaaxababaGagiyBa0MaeiyyaeMaeiiEaGhaleaacqaIXaqmcqGHKjYOcqWGPbqAcqGHKjYOcqWGobGtaeqaaaqaaiabdQgaQjabg2da9iabigdaXaqaaiabd2eanbqdcqGHris5aOGaem4Cam3aaSbaaSqaaiabdMgaPjabdQgaQbqabaGccqGGUaGlcaWLjaGaaCzcamaabmaabaGaeG4naCdacaGLOaGaayzkaaaaaa@59C6@

*rowScore *and *columnScore *lie in the interval [0, 1].

One alternative of combining the scores for both directions is to calculate their average. This scoring enforces that both gene products have the same types of functionality because a high score can only be achieved if *columnScore *and *rowScore *are high.

Another alternative is to compute the maximum of *rowScore *and *columnScore*:

*GOscore *= max{*columnScore*, *rowScore*},     (8)

where *GOscore *is the generic name for either *MFscore *if it is based on molecular function or *BPscore *if it is based on biological process. This score does not penalize situations where all GO terms of one gene product match a subset of the GO terms of the second gene product. This situation occurs when the annotation of the first gene product is not complete or when the second gene product is multi-functional.

### funSim

The *funSim *score is calculated from the *MFscore *and the *BPscore *of a pair of gene products. Two gene products with a high score in one ontology but only an average score in the other one can be considered average matches. However, their score should be higher than the score of two gene products that are average matches in both categories. Simply adding *MFscore *and *BPscore *or taking the average would not distinguish between these two cases. Squaring the *MFscore *and the *BPscore *favors high similarity in one ontology and a low score in the other one over average scores in both ontologies, thus allowing a distinction between these two scenarios. Therefore, the *funSim *score for two gene products is calculated as:

funSim=12⋅[(BPscoremax(BPscore))2+(MFscoremax(MFscore))2].     (9)
 MathType@MTEF@5@5@+=feaafiart1ev1aaatCvAUfKttLearuWrP9MDH5MBPbIqV92AaeXatLxBI9gBaebbnrfifHhDYfgasaacH8akY=wiFfYdH8Gipec8Eeeu0xXdbba9frFj0=OqFfea0dXdd9vqai=hGuQ8kuc9pgc9s8qqaq=dirpe0xb9q8qiLsFr0=vr0=vr0dc8meaabaqaciaacaGaaeqabaqabeGadaaakeaacqWGMbGzcqWG1bqDcqWGUbGBcqWGtbWucqWGPbqAcqWGTbqBcqGH9aqpdaWcaaqaaiabigdaXaqaaiabikdaYaaacqGHflY1daWadaqaamaabmaabaWaaSaaaeaacqWGcbGqcqWGqbaucqWGZbWCcqWGJbWycqWGVbWBcqWGYbGCcqWGLbqzaeaaieGacqWFTbqBcqWFHbqycqWF4baEcqGGOaakcqWGcbGqcqWGqbaucqWGZbWCcqWGJbWycqWGVbWBcqWGYbGCcqWGLbqzcqGGPaqkaaaacaGLOaGaayzkaaWaaWbaaSqabeaacqaIYaGmaaGccqGHRaWkdaqadaqaamaalaaabaGaemyta0KaemOrayKaem4CamNaem4yamMaem4Ba8MaemOCaiNaemyzaugabaGae8xBa0Mae8xyaeMae8hEaGNaeiikaGIaemyta0KaemOrayKaem4CamNaem4yamMaem4Ba8MaemOCaiNaemyzauMaeiykaKcaaaGaayjkaiaawMcaamaaCaaaleqabaGaeGOmaidaaaGccaGLBbGaayzxaaGaeiOla4IaaCzcaiaaxMaadaqadaqaaiabiMda5aGaayjkaiaawMcaaaaa@7726@

Here, *max*(*BPscore*) and *max*(*MFscore*) denote the maximum possible score for biological process and molecular function, respectively. If *sim*_*Rel *_is used, the *funSim *score lies in the interval [0, 1]. We use *sim*_*Rel *_for our analysis throughout the article.

Lin's similarity is not a metric since it does not satisfy the triangle inequality. This also holds for *funSim*. *funSim *can be applied to any type of gene product that is annotated with GO terms. Furthermore, it can be calculated with any semantic similarity measure that has a well-defined maximum.

### Derivation of the set IO

The set with Inparanoid orthologs (IO) was extracted from Inparanoid version 4.0 [34]. *Saccharomyces cerevisiae *proteins and human proteins with a score of 1.0 have been extracted from each Inparanoid cluster. All yeast-human protein pairs where both proteins had biological process and molecular function annotation were used. In total 682 protein pairs were obtained.

### Derivation of the sets LSS and HSS

For the two sets of protein pairs with low sequence similarity (LSS) and high sequence similarity (HSS), a BLAST [45,46] search of all yeast proteins from the IO set against all human proteins from Inparanoid was performed. All human sequences without biological process or molecular function annotation were filtered out. The proteins where mapped to UniProt using the ENSEMBL [47] BioMart tool on October 26th, 2005. All sequences without GO annotation were excluded. We mapped the SGD accession numbers of the yeast protein sequences to UniProt accession numbers with the UniProt 5.8 dat files. A BLAST comparison was carried out with version 2.2.12. Default parameters with an e-value threshold of 0.003 were used. The LSS data set contains for each yeast protein the human protein with the highest e-value that is not the ortholog. The human protein with the lowest e-value that is not the ortholog was included in the HSS dataset. Each of the two sets contains 989 protein pairs.

### Derivation of the set NSS

In order to compile a set of protein pairs with no sequence similarity (NSS), all human proteins with biological process and molecular function annotation that are not in the IO set were selected. One of these human proteins was assigned randomly to each yeast protein from the IO set. The proteins had no significant sequence similarity. The NSS set contains 1356 protein pairs.

### Comparison with Lord *et al*

We used the IO, HSS, LSS, and NSS datasets mentioned before for this analysis. The semantic similarity between single GO terms was calculated using the *sim*_*Rel *_measure. For the comparison of proteins, the *GOscore*_*Lord *_was computed according to the following formula:

GOscoreLord=1N∗M∑i=1N∑j=1Msij.     (10)
 MathType@MTEF@5@5@+=feaafiart1ev1aaatCvAUfKttLearuWrP9MDH5MBPbIqV92AaeXatLxBI9gBaebbnrfifHhDYfgasaacH8akY=wiFfYdH8Gipec8Eeeu0xXdbba9frFj0=OqFfea0dXdd9vqai=hGuQ8kuc9pgc9s8qqaq=dirpe0xb9q8qiLsFr0=vr0=vr0dc8meaabaqaciaacaGaaeqabaqabeGadaaakeaacqWGhbWrcqWGpbWtcqWGZbWCcqWGJbWycqWGVbWBcqWGYbGCcqWGLbqzdaWgaaWcbaGaemitaWKaem4Ba8MaemOCaiNaemizaqgabeaakiabg2da9maalaaabaGaeGymaedabaGaemOta4Kaey4fIOIaemyta0eaamaaqahabaWaaabCaeaacqWGZbWCdaWgaaWcbaGaemyAaKMaemOAaOgabeaaaeaacqWGQbGAcqGH9aqpcqaIXaqmaeaacqWGnbqta0GaeyyeIuoaaSqaaiabdMgaPjabg2da9iabigdaXaqaaiabd6eaobqdcqGHris5aOGaeiOla4IaaCzcaiaaxMaadaqadaqaaiabigdaXiabicdaWaGaayjkaiaawMcaaaaa@57D9@

This corresponds to the original definition form Lord *et al*. [16]. *MFscore*_*Lord *_and *BPscore*_*Lord *_correspond to the *GOscore*_*Lord *_for molecular function and biological process, respectively.

### Comparisons

We compared the biological processes from fungi to processes from mammals and the comparison of molecular functions from *Mycobacteria *to functions from mammals. The distributions for IO, HSS, LSS, and NSS where calculated using the *funSim *score. The *MFscore *and the *BPscore *were used to calculate the corresponding *GO score *distributions for the IO set. The comparison of yeast with human proteins was done with the *funSim *score. In this comparison, we used the 7356 yeast proteins and the 70 447 proteins from human from UniProt release 5.8. Almost 3 000 proteins could not be analyzed because there is no GO annotation available. Another 1 300 proteins have either no molecular function or no biological process assigned, giving an incomplete score. The data files for the comparison of biological processes from fungi and mammals ("bp_fungi_mammals.txt"), the comparison of molecular functions from *Mycobacteria *and mammals ("mf_myco_mammals.txt"), and the *funSim *comparison of yeast with human ("sc_hs.txt") are available for download at .

### Multidimensional scaling

The statistical software environment R () was used to perform metric multidimensional scaling (MDS). All yeast proteins with molecular function annotation were compared mutually yielding a square symmetric similarity matrix. Since the *MFscore *is a similarity measure and no distance, the distance of two proteins was computed as *d*_*mf *_= 1 - *MFscore*. The same procedure was applied to the molecular function annotation of the Pfam families. A square symmetric *d*_*mf *_matrix was used as input for the *cmdscale *method in R to perform a metric MDS. The normalized stress (NS) was calculated as

NS=∑ij(d′ij−dij)2∑ijdij2     (11)
 MathType@MTEF@5@5@+=feaafiart1ev1aaatCvAUfKttLearuWrP9MDH5MBPbIqV92AaeXatLxBI9gBaebbnrfifHhDYfgasaacH8akY=wiFfYdH8Gipec8Eeeu0xXdbba9frFj0=OqFfea0dXdd9vqai=hGuQ8kuc9pgc9s8qqaq=dirpe0xb9q8qiLsFr0=vr0=vr0dc8meaabaqaciaacaGaaeqabaqabeGadaaakeaacqWGobGtcqWGtbWucqGH9aqpdaWcaaqaamaaqababaWaaeWaaeaacuWGKbazgaqbamaaBaaaleaacqWGPbqAcqWGQbGAaeqaaOGaeyOeI0Iaemizaq2aaSbaaSqaaiabdMgaPjabdQgaQbqabaaakiaawIcacaGLPaaadaahaaWcbeqaaiabikdaYaaaaeaacqWGPbqAcqWGQbGAaeqaniabggHiLdaakeaadaaeqaqaaiabdsgaKnaaDaaaleaacqWGPbqAcqWGQbGAaeaacqaIYaGmaaaabaGaemyAaKMaemOAaOgabeqdcqGHris5aaaakiaaxMaacaWLjaWaaeWaaeaacqaIXaqmcqaIXaqmaiaawIcacaGLPaaaaaa@4F3E@

where d′ij
 MathType@MTEF@5@5@+=feaafiart1ev1aaatCvAUfKttLearuWrP9MDH5MBPbIqV92AaeXatLxBI9gBaebbnrfifHhDYfgasaacH8akY=wiFfYdH8Gipec8Eeeu0xXdbba9frFj0=OqFfea0dXdd9vqai=hGuQ8kuc9pgc9s8qqaq=dirpe0xb9q8qiLsFr0=vr0=vr0dc8meaabaqaciaacaGaaeqabaqabeGadaaakeaacuWGKbazgaqbamaaBaaaleaacqWGPbqAcqWGQbGAaeqaaaaa@30ED@ is the distance of proteins *i *and *j *in the low-dimensional space and *d*_*ij *_the respective distance in the original space. The change rate of normalized stress (CR) was calculated as

CRk=(NSk−NSk−1)(NSk+1−NSk)     (12)
 MathType@MTEF@5@5@+=feaafiart1ev1aaatCvAUfKttLearuWrP9MDH5MBPbIqV92AaeXatLxBI9gBaebbnrfifHhDYfgasaacH8akY=wiFfYdH8Gipec8Eeeu0xXdbba9frFj0=OqFfea0dXdd9vqai=hGuQ8kuc9pgc9s8qqaq=dirpe0xb9q8qiLsFr0=vr0=vr0dc8meaabaqaciaacaGaaeqabaqabeGadaaakeaacqWGdbWqcqWGsbGudaWgaaWcbaGaem4AaSgabeaakiabg2da9maalaaabaGaeiikaGIaemOta4Kaem4uam1aaSbaaSqaaiabdUgaRbqabaGccqGHsislcqWGobGtcqWGtbWudaWgaaWcbaGaem4AaSMaeyOeI0IaeGymaedabeaakiabcMcaPaqaaiabcIcaOiabd6eaojabdofatnaaBaaaleaacqWGRbWAcqGHRaWkcqaIXaqmaeqaaOGaeyOeI0IaemOta4Kaem4uam1aaSbaaSqaaiabdUgaRbqabaGccqGGPaqkaaGaaCzcaiaaxMaadaqadaqaaiabigdaXiabikdaYaGaayjkaiaawMcaaaaa@4ED3@

with *k *being the number of dimensions. Densities have been estimated with a two-dimensional Gaussian kernel estimation by the kde2d function from the R software.

### Hierarchical clustering

The hierarchical clustering was done with Pycluster version 1.29 () and Python 2.4.2 () using a maximum linkage clustering algorithm. The distance matrix was the same as used for the MDS.

## Authors' contributions

AS developed the method under the supervision of FD and TL. JR contributed to the development of the method. AS and FD evaluated and interpreted the results. Every author contributed to the final version of the paper.

## Supplementary Material

Additional file 1 **Detailed results from sections "Comparing biological processes and molecular functions" and "Comparison of *funSim *and sequence similarity"**. Table S1: The 50 biological processes from fungi with lowest *sim*_*Rel *_values compared to mammalian processes. Table S2: The molecular functions from *Mycobacterium *with lowest *sim*_*Rel *_values compared to mammalian functions. Figure S1: Distribution of the *MFscore *(A), *BPscore *(B), *funSim *score (C) for different sets of protein pairs using GO annotation with all evidence codes. Click here for file
