# Supplementary material for: A new measure for functional similarity of gene products based on Gene Ontology
Source: BMC Bioinformatics. 2006 Jun 15;7:302. doi: 10.1186/1471-2105-7-302 (PMC1559652; doi:10.1186/1471-2105-7-302)
Supplement: Additional file 1 — Detailed results from sections "Comparing biological processes and molecular functions" and "Comparison of funSim and sequence similarity". Table S1: The 50 biological processes from fungi with lowest simRel values compared to mammalian processes. Table S2: The molecular functions from Mycobacterium with lowest simRel values compared to mammalian functions. Figure S1: Distribution of the MFscore (A), BPscore (B), funSim score (C) for different sets of protein pairs using GO annotation with all evidence codes. [file 1471-2105-7-302-S1.pdf]

Supplemental Data
A new Measure for functional Similarity of Gene Products based on Gene Ontology

Andreas Schlicker[1*], Francisco S. Domingues[1] , Jörg Rahnenführer[1] , Thomas Lengauer[1]

[1]Department of Computational Biology and Applied Algorithmics, Max-Planck-Institute for Informatics, Stuhlsatzenhausweg 85, 66123 Saarbr ̈ucken, Germany

Email: Andreas Schlicker*- andreas.schlicker@mpi-inf.mpg.de; Francisco S. Domingues - doming@mpi-inf.mpg.de; Jörg Rahnenf ̈uhrer - rahnenfj@mpi-inf.mpg.de; Thomas Lengauer - lengauer@mpi-inf.mpg.de;

*Corresponding author

Supplement
Comparing biological processes and molecular functions

Table S1: The 50 biological processes from fungi with lowest sim_{Rel} values compared to mammalian processes.

acc	name	sim_{Rel}	acc
GO:0030541	plasmid partitioning	0.15808	GO:0006278
GO:0006033	chitin localization	0.30027	GO:0015986
GO:0046713	boron transport	0.31932	GO:0015986
GO:0009302	snoRNA transcription	0.38639	GO:0006367
GO:0006089	lactate metabolism	0.38782	GO:0009082
GO:0019630	quinate metabolism	0.39903	GO:0009082
GO:0019541	propionate metabolism	0.42775	GO:0009082
GO:0042128	nitrate assimilation	0.4502	GO:0009082
GO:0009305	protein amino acid biotinylation	0.4587	GO:0006470
GO:0016926	protein desumoylation	0.47222	GO:0006470
GO:0031144	proteasome localization	0.4916	GO:0006614
GO:0045116	protein neddylation	0.49535	GO:0006470
GO:0000338	protein deneddylation	0.51801	GO:0006470
GO:0006279	premeiotic DNA synthesis	0.52348	GO:0006278
GO:0006522	alanine metabolism	0.53138	GO:0009082
GO:0019985	bypass DNA synthesis	0.53997	GO:0006303
GO:0048309	endoplasmic reticulum inheritance	0.54037	GO:0006364
GO:0015847	putrescine transport	0.55201	GO:0015813
GO:0031291	Ran protein signal transduction	0.55494	GO:0046580
GO:0015801	aromatic amino acid transport	0.55565	GO:0015813
GO:0042762	regulation of sulfur metabolism	0.55802	GO:0009086
GO:0045458	recombination within rDNA repeats	0.55879	GO:0006313
GO:0018298	protein-chromophore linkage	0.56308	GO:0006470
GO:0000256	allantoin catabolism	0.56748	GO:0006783
GO:0040031	snRNA modification	0.58001	GO:0006437
GO:0042545	cell wall modification	0.58265	GO:0006364
GO:0000373	Group II intron splicing	0.58438	GO:0006388
GO:0000358	formation of catalytic U2-type spliceosome for second transesterification step	0.58438	GO:0006388

Table S1: The 50 biological processes from fungi with lowest sim_{Rel} values compared to mammalian processes.

acc	name	sim_{Rel}	acc
GO:0000396	U2-type spliceosome conformational change to release U4 and U1	0.58438	GO:0006388
GO:0046459	short-chain fatty acid metabolism	0.58763	GO:0006636
GO:0001718	conversion of met-tRNAf to fmet-tRNA	0.58925	GO:0006437
GO:0007116	regulation of cell budding	0.58973	GO:0008356
GO:0030582	fruiting body formation	0.59246	GO:0007309
GO:0030994	primary cell septum hydrolysis	0.59514	GO:0000921
GO:0030995	cell septum edging hydrolysis	0.59514	GO:0000921
GO:0006425	glutaminyl-tRNA aminoacylation	0.59627	GO:0006437
GO:0043007	rDNA maintenance	0.60271	GO:0007076
GO:0015729	oxaloacetate transport	0.60369	GO:0015744
GO:0015741	fumarate transport	0.60369	GO:0015744
GO:0006513	protein monoubiquitination	0.60451	GO:0000209
GO:0000356	U2-type catalytic spliceosome formation for first transesterification step	0.61587	GO:0006388
GO:0017062	cytochrome bc(1) complex assembly	0.61894	GO:0008535
GO:0000372	Group I intron splicing	0.62573	GO:0006388
GO:0046482	para-aminobenzoic acid metabolism	0.62785	GO:0046653
GO:0006315	homing of group II introns	0.62954	GO:0006313
GO:0006322	Ty3 element transposition	0.62954	GO:0006313
GO:0006320	Ty1 element transposition	0.62954	GO:0006313
GO:0006316	movement of group I intron	0.62954	GO:0006313
GO:0031321	prospore formation	0.63094	GO:0007595
GO:0015772	oligosaccharide transport	0.63182	GO:0009401

Table S2: The molecular functions from *Mycobacterium* with lowest sim_{Rel} values compared to mammalian functions.

acc	name	sim_{Rel}	acc
GO:0008686	3&4-dihydroxy-2-butanone-4-phosphate synthase activity	0.05293	GO:0003968
GO:0018786	haloalkane dehalogenase activity	0.13931	GO:0046961
GO:0004125	L-seryl-tRNASec selenium transferase activity	0.18767	GO:0003968
GO:0043365	[formate-C-acetyltransferase]-activating enzyme	0.30076	GO:0008137
GO:0008862	formate acetyltransferase activating enzyme activity	0.3383	GO:0008137
GO:0016216	isopenicillin-N synthase activity	0.35215	GO:0008137
GO:0004475	mannose-1-phosphate guanylyltransferase activity	0.3757	GO:0003968
GO:0008773	[protein-PII] uridylyltransferase activity	0.39112	GO:0003968
GO:0003919	FMN adenylyltransferase activity	0.39503	GO:0003968
GO:0050348	trehalose O-mycolyltransferase activity	0.40932	GO:0004316
GO:0004654	polyribonucleotide nucleotidyltransferase activity	0.4118	GO:0003968
GO:0047330	polyphosphate-glucose phosphotransferase activity	0.4182	GO:0000155
GO:0016210	naringenin-chalcone synthase activity	0.4216	GO:0004316
GO:0030401	transcription antiterminator activity	0.42698	GO:0000156
GO:0008910	kanamycin kinase activity	0.42998	GO:0000155
GO:0008928	mannose-1-phosphate guanylyltransferase (GDP) activity	0.43487	GO:0003968
GO:0008879	glucose-1-phosphate thymidylyltransferase activity	0.43877	GO:0003968
GO:0008710	8-amino-7-oxononanoate synthase activity	0.46909	GO:0004316
GO:0016852	sirohydrochlorin cobaltochelatase activity	0.48446	GO:0004730
GO:0008968	phosphoheptose isomerase activity	0.51468	GO:0003918
GO:0008887	glycerate kinase activity	0.52137	GO:0000155
GO:0016851	magnesium chelatase activity	0.52842	GO:0004842
GO:0004063	aryldialkylphosphatase activity	0.52996	GO:0008967
GO:0000036	acyl carrier activity	0.53992	GO:0046961
GO:0046025	precorrin-6Y C5&15-methyltransferase (decarboxylating) activity	0.55599	GO:0008898
GO:0046026	precorrin-4 C11-methyltransferase activity	0.56021	GO:0008898
GO:0008832	dGTPase activity	0.57983	GO:0008967
GO:0008691	3-hydroxybutyryl-CoA dehydrogenase activity	0.58093	GO:0004616
GO:0045156	electron transporter& transferring electrons within the cyclic electron transport pathway of photosynthesis activity	0.58192	GO:0008137
GO:0008949	oxalyl-CoA decarboxylase activity	0.5918	GO:0004638
GO:0008762	UDP-N-acetylmuramate dehydrogenase activity	0.59661	GO:0004616
GO:0008999	ribosomal-protein-alanine N-acetyltransferase activity	0.59692	GO:0004742
GO:0008825	cyclopropane-fatty-acyl-phospholipid synthase activity	0.59789	GO:0008898
GO:0004190	aspartic-type endopeptidase activity	0.60033	GO:0004194
GO:0008945	oligopeptidase B activity	0.6088	GO:0004263
GO:0008810	cellulase activity	0.61968	GO:0004308
GO:0008124	4-alpha-hydroxytetrahydrobiopterin dehydratase activity	0.6278	GO:0004730
GO:0008677	2-dehydropantoate 2-reductase activity	0.6295	GO:0004616
GO:0008767	UDP-galactopyranose mutase activity	0.63708	GO:0004619
GO:0004401	histidinol-phosphatase activity	0.63717	GO:0008967
GO:0004412	homoserine dehydrogenase activity	0.6411	GO:0004616
GO:0016993	precorrin-8X methylmutase activity	0.64821	GO:0004619
GO:0004015	adenosylmethionine-8-amino-7-oxononanoate transaminase activity	0.6486	GO:0004084
GO:0042286	glutamate-1-semialdehyde 2&1-aminomutase activity	0.65019	GO:0004619
GO:0004764	shikimate 5-dehydrogenase activity	0.65187	GO:0004616
GO:0008703	5-amino-6-(5-phosphoribosylamino)uracil reductase activity	0.65523	GO:0004616
GO:0050525	cutinase activity	0.65685	GO:0004806

Table S2: The molecular functions from *Mycobacterium* with lowest sim_{Rel} values compared to mammalian functions.

acc	name	sim_{Rel}	acc
GO:0008909	isochorismate synthase activity	0.65839	GO:0004619
GO:0047575	4-carboxymuconolactone decarboxylase activity	0.66286	GO:0004638
GO:0003961	O-acetylhomoserine aminocarboxypropyltransferase activity	0.66414	GO:0004156
GO:0018492	carbon-monoxide dehydrogenase (acceptor) activity	0.66437	GO:0004365
GO:0008806	carboxymethylenebutenolidase activity	0.66481	GO:0004806
GO:0004160	dihydroxy-acid dehydratase activity	0.67188	GO:0004730
GO:0017168	5-oxoprolinase (ATP-hydrolyzing) activity	0.67685	GO:0050480
GO:0008939	nicotinate-nucleotide-dimethylbenzimidazole phosphoribosyltransferase activity	0.67886	GO:0004731
GO:0008683	2-oxoglutarate decarboxylase activity	0.68297	GO:0004638
GO:0048307	ferredoxin-nitrite reductase activity	0.68665	GO:0004846
GO:0005351	sugar porter activity	0.68778	GO:0015520
GO:0003861	3-isopropylmalate dehydratase activity	0.69434	GO:0004730
GO:0008774	acetaldehyde dehydrogenase (acetylating) activity	0.69537	GO:0004365
GO:0004124	cysteine synthase activity	0.69717	GO:0004730
GO:0004106	chorismate mutase activity	0.69748	GO:0004619
GO:0004748	ribonucleoside-diphosphate reductase activity	0.69767	GO:0051061

Comparison of *funSim* and sequence similarity

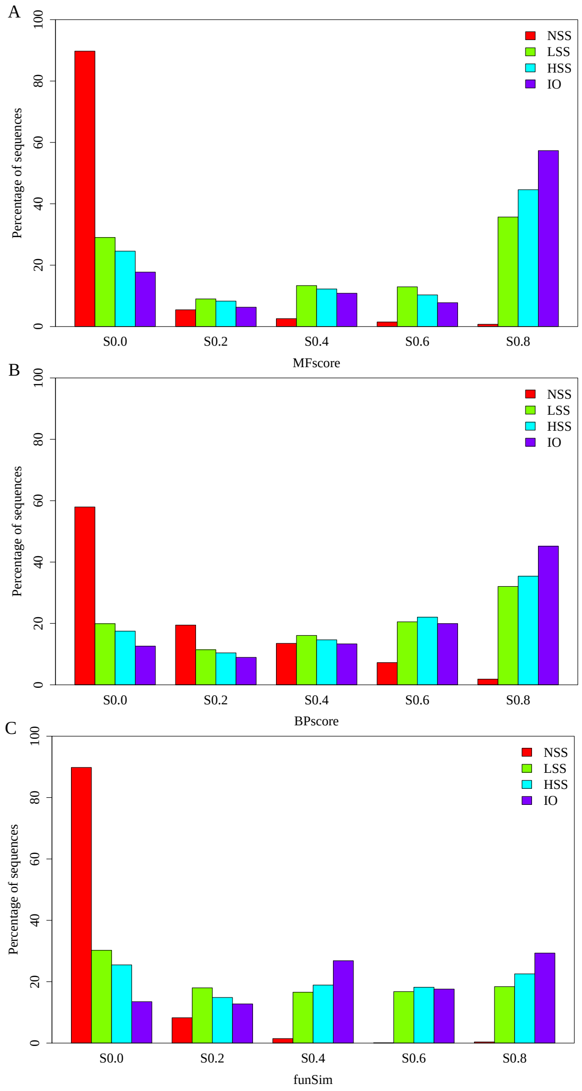
A
100
80
60
40
20
0
Percentage of sequences
NSS
LSS
HSS
IO
S0.0
S0.2
S0.4
S0.6
S0.8
MFscore
B
100
80
60
40
20
0
Percentage of sequences
NSS
LSS
HSS
IO
S0.0
S0.2
S0.4
S0.6
S0.8
BPscore
C
100
80
60
40
20
0
Percentage of sequences
NSS
LSS
HSS
IO
S0.0
S0.2
S0.4
S0.6
S0.8
funSim

Figure S1 - Distribution of the $MFscore$ (A), $BPscore$ (B), $funSim$ score (C) for different sets of protein pairs. The bins correspond to the following intervals of $funSim$ values: S0.0: $[0.0, 0.2[$; S0.2: $[0.2, 0.4[$; S0.4: $[0.4, 0.6[$; S0.6: $[0.6, 0.8[$; S0.8: $[0.8, 1.0]$. The percentage values are calculated according to the total number of protein pairs in the different categories. The sets of the different categories contain the following numbers of protein pairs: NSS 1356, LSS and HSS 989 each, and IO 682. The derivation of the sets is explained in the Materials and Methods section.
